# unDerstandIng the cauSes of mediCation errOrs and adVerse drug evEnts for patients with mental illness in community caRe (DISCOVER): a qualitative study

**DOI:** 10.3389/fpsyt.2023.1241445

**Published:** 2023-12-07

**Authors:** Matthew J. Ayre, Penny J. Lewis, Denham L. Phipps, Richard N. Keers

**Affiliations:** ^1^Division of Pharmacy and Optometry, School of Health Sciences, Faculty of Biology, Medicine and Health, The University of Manchester, Manchester, United Kingdom; ^2^NIHR Greater Manchester Patient Safety Translational Research Centre, Manchester Academic Health Science Centre (MAHSC), The University of Manchester, Manchester, United Kingdom; ^3^Manchester University NHS Foundation Trust, Manchester, United Kingdom; ^4^Optimising Outcomes with Medicines (OptiMed) Research Unit, Pennine Care NHS Foundation Trust, Manchester, United Kingdom

**Keywords:** mental disorder, psychiatry, primary health care, community mental health services, medication error, adverse drug event

## Abstract

**Background:**

It is estimated that 237 million medication errors occur in England each year with a significant number occurring in the community. Our understanding of the causes of preventable medication errors and adverse drug events (ADE) affecting patients with mental illness is limited in this setting. Better understanding of the factors that contribute to errors can support the development of theory-driven improvement interventions.

**Methods:**

Remote qualitative semi-structured interviews with 26 community-based healthcare professionals in England and Wales were undertaken between June–November 2022. Recruitment was undertaken using purposive sampling via professional networks. Interviews were guided by the critical incident technique and analysed using the framework method. Any data that involved speculation was not included in the analysis. Independent analysis was carried out by the research team to extract themes guided by the London Protocol.

**Results:**

A total of 43 medication errors and 12 preventable ADEs were discussed, with two ADEs having an unknown error origin. Prescribing errors were discussed most commonly (*n* = 24), followed by monitoring errors (*n* = 8). Six contributory factor themes were identified: the individual (staff); the work environment; the teams/interfaces; the organisation and management; the patient; and the task and technology. The individual (staff) factors were involved in just over 80% of all errors discussed. Participants reported a lack of knowledge regarding psychotropic medication and mental illnesses which accompanied diffusion of responsibility. There were difficulties with team communication, particularly across care interfaces, such as ambiguity/brevity of information being communicated and uncertainty concerning roles which created confusion amongst staff. Unique patient social/behavioural contributory factors were identified such as presenting with challenging behaviour and complex lifestyles, which caused difficulties attending appointments as well as affecting overall clinical management.

**Conclusion:**

These findings highlight that the causes of errors are multifactorial with some unique to this patient group. Key areas to target for improvement include the education/training of healthcare professionals regarding neuropharmacology/mental illnesses and enhancing communication across care interfaces. Future research should explore patient perspectives regarding this topic to help develop a holistic picture. These findings can be used to guide future intervention research to ameliorate medication safety challenges for this patient group.

## Introduction

Medication safety and mental health patient safety have become key healthcare priorities within the National Health Service (NHS) in the United Kingdom (UK) (1). An estimated 237 million medication errors occur in England each year with 38.4% occurring in the community (2). Medication errors and adverse drug events (ADE) are one of the leading causes of death in healthcare and can increase healthcare expenditure (2, 3). A review and meta-analysis examined preventable medication harm and found that this was most prevalent in central nervous system drugs of which psychotropics belong (4). There can be additional complexities with the management of medication for patients with mental illness such as polypharmacy, inappropriate prescribing and anosognosia (5–9). A recent scoping review highlighted that patients with mental illness commonly experience a variety of medication safety challenges in the community such as non-adherence, potentially inappropriate prescribing, medication errors and ADEs (10). The review also highlighted the causes and risk factors for some of these issues; however, these causal data mostly related to non-adherence with little data for medication errors and preventable ADEs (10). Understanding this research gap regarding the causes of these preventable events will aid with the development of effective theory-driven interventions (11, 12) to improve medication safety in this group of patients.

Around 90.0% of patients with mental illness are treated solely within the community (13) and 40.0% of general practitioner (GP) appointments may concern mental health (14). Community care settings are integral to the prescribing, monitoring and dispensing of psychotropic drugs (15–18), and this sector therefore has a pivotal role in the care and supply of medication for patients with mental illness. Over recent years there has been increased research activity exploring medication safety concerns in secondary care mental health settings (19–24), with patient and system wide risk factors and contributors identified such as patient behaviours, impaired cognition (23, 25), lack of continuity of care and communication issues (20, 23). However, contributors identified from secondary care may not be applicable to the community (23) and therefore may hinder development of theory-driven remedial interventions in this setting. This study therefore aims to address the research needs identified above through a qualitative exploration to understand in-depth what factors contribute to medication errors and preventable ADEs for patients with mental illness in the community.

## Materials and methods

### Study design and setting

This study used a qualitative design and the setting was community care in England and Wales. This included settings such as general practice, community pharmacy, community mental health services, primary care networks (collaborative/integrated healthcare) (26), district nursing services, nursing/care homes and substance misuse services (27).

### Definitions

In this study medication errors were defined as “*any preventable event that may cause or lead to inappropriate medication use or patient harm while the medication is in the control of the health care professional, patient, or consumer”* (28). Events were categorised relating to the domains of medication prescribing, monitoring, dispensing and administration. ADEs were defined as an injury due to the use of a medication (29) and considered preventable if best medical practice was not adhered to (30, 31). Serious mental illness was defined as an illness with substantial functional impairment and included non-organic psychotic disorders, major affective disorder and personality disorders (32). Less serious mental illness was any other mental illness falling outside of the serious mental illness definition.

### Patient and public involvement and engagement

Four individuals with experience of using mental health services or caring for someone in mental health services were consulted on the design of the study and helped refine the interview schedule. Another patient advisory group of three expert patients with lived experience of mental illness were consulted three times throughout the duration of the study in order to provide advice regarding recruitment, validation of identified themes and their interpretation.

### Participants and recruitment

The sampling frame comprised of pharmacists, GPs, nurses, psychiatrists and substance misuse specialists with experience of working in a UK-based community service within the last 5 years. Within this frame, participants were recruited via purposive sampling to represent a range of professional roles and community settings. We sought participants who had first-hand experience of dealing with medication errors in relation to patients with mental illness (33). Participants were approached through the research team’s regional and national professional networks or through social media advertisements between June–November 2022. Snowballing was encouraged to reach potential participants that the research team did not have direct access to. Participants who expressed interest to take part were then asked to complete a signed consent form before being eligible to attend the interview. Interviews were conducted until data saturation was met which was predicted to be ~20 interviews, as little new information is generated beyond this number (34). Interviews continued beyond this target to further validate and strengthen the data given the heterogeneity of the professional groups.

### Data collection

Interviews were conducted remotely via Zoom^™^. The interview guide (Supplementary file 1) was adapted from a study of medication administration errors using the critical incident technique (CIT) in secondary care mental health services (20) before being refined through consultation with the patient advisory group and piloting. The CIT enables exploration of the processes leading up to an incident and the consequences (35) and has already been applied to medication error and health research in other settings (20, 36–38). The CIT also reduces the propensity of interviewees to provide generic answers by encouraging them to focus on specific details of an incident (35). The interview consisted of first gathering information about the participant such as professional/training background, then a detailed exploration of one or more medication errors and/or preventable ADEs that the participant had made or that had direct first-hand knowledge of including the origin of the error(s) and the reasons for its occurrence. Finally, the participant discussed how they believed these incidents could have been avoided. Participants were encouraged to discuss errors that had happened recently or that could be recalled clearly. MJA conducted the interviews using a semi-structured interview guide. All interviews were video/audio recorded and sent to a professional university approved transcription company to undergo intelligent verbatim transcription. Returned transcripts were then checked against the original recording for accuracy by the main researcher (MJA). The interview length ranged between 23 and 91 minutes with the average length being 50.3 minutes.

### Data analysis

Interview transcripts were uploaded into NVivo 12 Plus software for analysis using the framework method (39). This method allows systematic reduction of the data and in-depth analysis of key themes across the entire dataset without losing context (39) and has been used in other health research examining the causes of medication errors (20, 38). The framework method included seven steps: transcribing; reading and becoming familiar with the interviews; specific descriptive codes; developing a framework by grouping into established themes; applying the framework to the remainder of the interviews; charting the data into matrices; and finally interpreting the data. Participants were instructed in the participant information sheet to provide only first-hand accounts. Participant accounts that were based on speculation (i.e. no first-hand experience of the error) about the causes of errors/ADEs were excluded from any analysis. Independent analysis of the transcripts was carried out by the research team (MJA, RNK, PJL and DLP) who then all met to reach consensus, in order to help validate the themes generated. Consultation was carried out with the research team and the patient advisory group regarding the themes that were generated with an iterative process following to refine the data. The data were organised into themes based on the London Protocol (40) which builds upon Reason’s error framework (41), and has been used in other medication error research in community settings (42). The London Protocol proposes an analysis of clinical incidents by placing contributory factors into seven domains: patient; task and technology; individual (staff); team; work environment; organisation and management; and institutional (40).

### Ethical consideration and approval

Participants provided informed written consent to take part in this study. Participants were also given an interview ID to anonymise their contributions. Participants were being asked to recall preventable medication safety incidents which some may have found distressing, therefore, a distress protocol was developed as a precaution. This study was approved by the University of Manchester Research Ethics Committee 1 (reference 2022-13735-23555).

## Results

In total, 26 participants were interviewed from community care (14 pharmacists, 5 GPs, 5 nurses, 2 psychiatrists). A total of 45 medication incidents were discussed; 43 of these were medication errors, 10 involving a preventable ADE. An additional two ADEs were discussed with no reported origin specific to a stage of the medication use process. ADEs were deemed to have been preventable based on the participants’ considering the definition (see Methods) and making a judgement. The rates of incidents for patients with less serious mental illness were 20/45, followed by patients with serious mental illness (18/45) and 7/45 accounts could not recall/were unaware of the patients diagnosis at the time of interview. Prescribing errors were discussed most commonly (*n* = 24), followed by monitoring (*n* = 8), dispensing (*n* = 7) and administration (*n* = 4) errors. A summary of the numbers of each healthcare professional and which errors were discussed from which setting are displayed in Table 1.

**Table 1:** Summary of the number of professionals, settings and errors/ADEs.

Profession (total number)	Independent prescriber	Community care setting (number of professionals)	Prescribing (n) [ADE n]	Monitoring (n) [ADE n]	Dispensing (n) [ADE n]	Administration (n) [ADE n]	ADE with no reported origin (n)
Pharmacist (14)	5	General Practice/PCN (5)	8 [1]	2	0	1 [1]	–
		Community mental health services (3)	4 [1]	0	1	2 [1]	–
		Community pharmacy (6)	4 [1]	0	4 [1]	0	2
General Practitioner (5)	5	General Practice (5)	4 [2]	4	0	0	–
Nurse (5)	3	Community mental health services (5)	3 [2]	2	1	1	–
Psychiatrist (2)	2	Community mental health services (2)	1	0	1	0	–

ADE, Adverse drug event; PCN, Primary care network.

Participants varied in the number of years they had been qualified for that role with eight qualified for 1–3 years; one for 3–5 years; two for 5–10 years; nine for 10–20 years; and six for >20 years. The number of years practicing in a community role also varied with 11 practicing for 1–3 years; four for 3–5 years; two for 5–10 years; five for 10–20 years; and four for >20 years. A total of 24 participants were practicing in England and two participants were practicing in Wales. The majority of participants were practicing in Northwest England (17 participants). Analysis of coded error accounts from participants of differing years of experience and from different geographical locations, found no observable differences in their reported contributory factors. In total, 6 errors were made by the participant being interviewed and the remaining 39 errors were made by another clinician/colleague. There was no observable difference between self-made or colleague error accounts, so all data was analysed in the same way. This exploratory study found the London Protocol was an appropriate guide to assist with the extraction of contributory factor themes. A summary of the participant characteristics and the type of medication errors discussed with the contributory factors are displayed in Supplementary file 2.

### Contributory factors

**Figure 1:**
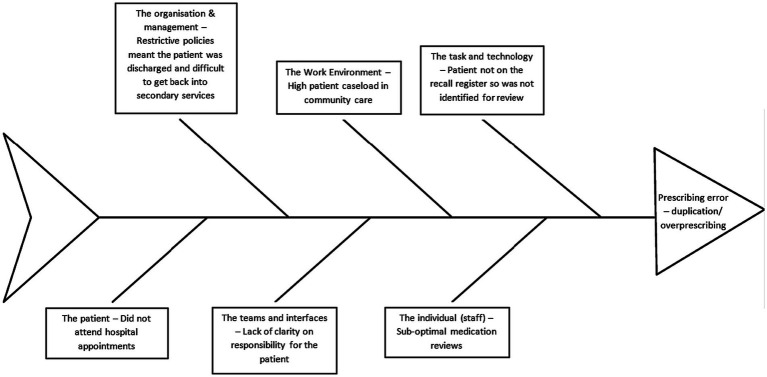
Fishbone diagram highlighting the contributory factors that led to a duplication/overprescribing error – *PHAR11* account. Adapted from the London Protocol (40).

The most common contributory factor discussed was the individual (staff) which was involved in a total of 37 errors/ADEs, followed by the work environment (*n* = 31) and teams and interfaces (*n* = 28). A list of the contributory factors and how many were implicated in each error type are displayed in Table 2. A full breakdown of the contributory factors by sub-themes are displayed in Supplementary file 3. Figure 1 demonstrates how multiple factors contributed to one specific error from one of the participant accounts. The main difference between error accounts across community care services were the types of error(s) that professionals focused on for discussion, for example, GPs focused solely on prescribing and monitoring events.

**Table 2:** Summary of the number of incidents each contributory factor was cited.

Factor	Prescribing [*n* = 24]	Monitoring [*n* = 8]	Dispensing [*n* = 7]	Administration [*n* = 4]	ADEs unknown origin [*n* = 2]	Total number of medication incidents
The individual (staff)	21	7	5	3	1	37
The work environment	18	4	6	3	–	31
The teams and interfaces	17	5	4	2	–	28
The organisation and management	12	6	5	1	–	24
The patient	13	6	–	2	2	23
The task and technology	9	5	1	–	–	15

ADE, Adverse drug event.

### The individual (staff)

Over half of the participants described a lack of knowledge, skills and training amongst community clinicians with regards to mental illnesses, psychotropic medication and of local shared care procedures (transfer of clinical responsibility from hospital to community). This was reported to be caused by unmet training needs but was also linked to a perceived lack of ownership and responsibility clinicians felt they had for what they considered to be a specialist patient group. One GP, when describing the origins of a prescribing error, described this relationship between familiarisation with psychotropics and responsibility:

*“…primary care [community] are less familiar with [antipsychotics] because [GPs] do not initiate them. So [we] spend less time checking in the BNF [British National Formulary], looking at doses, it’s more of a case [that] we are recommended [to start] them or [continue] them by specialists….”* *(GP04, General practice, qualified 1–3 years)*

This GP then went on to add that this lack of detailed knowledge of psychotropics created a barrier to community clinicians conducting independent scrutiny of medication, a view also highlighted by a minority of other participants. This was exemplified by their colleague reporting a practice of leaving other clinicians’ work even when there may be concerns, which contributed to inappropriate prescribing:

*“[The GP] said, oh, you know, who am I to question what patients should take?”* *(GP04, General practice, qualified 1–3 years)*

Healthcare professionals working in non-specialist community settings reported managing the care of patients with mental illness, yet they also viewed psychiatry as being outside of their remit of responsibility; this belief influenced the subtle decision to not develop more detailed knowledge of the medications involved. This was reported amongst cases involving patients with both serious and less serious mental illness.

A minority described psychiatric practice as *“paternalistic”* (NURSE01) in nature which was reported to generate a power imbalance between patients and clinicians and may also extend to clinician–clinician relationships. Some participants explained how this power dynamic could result in exaggeration of symptoms in order to validate their prescribing decisions, which contributed to inappropriate prescribing:

*“They [nursing staff] exaggerated the symptoms to the GP. The GP did not do a face to face visit. They [GP] provided a prescription over the phone.”* *(NURSE03, Community mental health services, qualified 10–20 years)*

### The work environment

The majority of participants discussed workload, time and shift pattern issues. Participants all described how service demand was exceeding the capacity it was designed to handle and how an increased workload was not matched by an increase in staffing. High workloads meant staff reported not having the time to work carefully, communicate with each other effectively or undergo necessary training, which contributed to them making errors because they were feeling stressed and distracted. One specialist pharmacist described how these factors resulted in inappropriate prescribing from clinicians working in a specialist service:

“*…because of the high workload we [GPs and pharmacists] were not able to just stop and say, hold on a second this [prescription] does not look right. We just did not have that time because we had so much to do*.” *(PHAR07, Community mental health services, qualified 5–10 years)*

A minority of participants described how workplaces were struggling to recruit as well as retain staff. Absent staff due to sickness, holidays or training left the remaining staff with a higher workload. As a result, participants described relying on agency/locum staff and staggered shift patterns, affecting the skill mix within the team and negatively impacting continuity of care. One locum community pharmacist described how a lack of continuity and variable shift patterns impacted their ability to gather all of the necessary information, resulting in an omission dispensing error:

*“There was no continuity. It will often be quite hard to get information because the response will be, I wasn’t there or I was only in a couple of hours. It did make it quite hard*.*” (PHAR06, Community pharmacy, qualified 10–20 years)*

Nearly a quarter of participants discussed the design of clinical resources, protocols and drug names specific to psychiatry as contributors to error. Clinical resources and protocols regarding shared care (defined earlier) were reported to be unclear and confusing, causing staff to misinterpret them or have difficulty locating the required information. For example, one general practice pharmacist highlighted that it can be difficult locating what information should be included in shared care agreements due to poor organisation of the website resources. The perceived similar sounds of drugs such as *“tramadol/trazodone”* or *“zuclopenthixol acetate/decanoate”* caused confusion as illustrated by one GP’s account of an error, when promazine was prescribed instead of promethazine:

*“The prescribing clerk read that and actually prescribed promazine, the antipsychotic instead. So two similar sounding, similar looking drugs, and on electronic prescribing I believe that when they put in P-R-O-M and then it auto-filled the drug choices they said that there was one just above the other, so they clicked the wrong one….” (GP04, General practice, qualified 1–3 years)*

### Teams and interfaces

Verbal and written communication between specialist mental health teams, general practices and community pharmacies was discussed by over half of participants as an important contributor to medication errors. Specifically, they described how it was difficult to communicate with other clinicians due to a lack of contact details and delays in receipt of clinical letters/information between services - this led to mainly prescribing and monitoring errors. Participants also described the lack of clinical updates and the absence or ambiguity/brevity of the clinical information that was communicated. This led to uncertainty in how to act due to being unaware of critical details that may have otherwise influenced their decisions. Participants highlighted that there could also be brief, as well as absent, verbal and written clinical information from the clinician to the patient. A general practice pharmacist described how a letter from specialist mental health services did not highlight that baseline physical health monitoring had not taken place resulting in a monitoring error:

*“The handwritten letter was just like a blank page and it just said, please can you start quetiapine MR 50 mg once a day, something like that, and just signed by the consultant. And that just did not have any information about the shared care protocol, anything like that*.*” (PHAR13, General practice/PCN, qualified 10–20 years)*

Over a quarter of participants discussed the ideas of team structure, inclusion and appropriate feedback loops. Participants described the increasing range of healthcare professionals working in community care services and the complexity this brings to the healthcare system. One GP described the team structure within their practice and how this had changed over the years which created a skill mix within the team (as described earlier), with each professional discipline having variable knowledge regarding practice procedures. These contributors impacted continuity of care and team skill mix which ultimately led to a prescription for an inappropriate indication:

*“In those twenty years, we have now gone from a practice of eighteen hundred patients to twenty-three thousand patients, we have got, how many GPs, ten plus locums, we have got nurse practitioners, practice nurses, healthcare assistants, a paramedic, pharmacists, pharmacy technicians, have we got anybody else, I do not know, that’s quite enough though, isn’t it. So, the complexity of the practice, it’s just changed out of all recognition and I think for me, there are some things about being a GP, like continuity of care, maintaining that relationship with people, having some control over prescribing, knowing what’s going on is important*.*” (GP03, General practice, qualified > 20 years)*

Some pharmacists reported that they felt excluded from the healthcare working environment and this was exacerbated by the healthcare system placing emphasis on particular roles such as GPs. Ultimately, this was reported to result in clinicians working independently without knowing the defined roles of colleagues and was linked to diffusion of responsibility which left the patient in *“no man’s land.”* (GP04) One general practice pharmacist outlined how they felt excluded from carrying out medication related tasks. The exclusion of their expertise contributed to a colleague’s omission and duplication prescribing error as the pharmacist was not able to provide input into the clinical scenario:

*“They’re [PCN pharmacy team] not really felt as if they are part of the surgery team…they should still have pharmacy technicians and pharmacists to do medicine reconciliation [inpatient settings]. But on a community outpatient […] you are relying on the psychiatrist or the prescriber that you see to keep that up to date because no one is going to have that GP system access that they would if you were in, either in perhaps hospital in mental health or general hospital. You would expect the same level of oversight and pharmacy support to go into that reconciling process. But because it’s community… [pharmacists] do not have that oversight*.*” (PHAR11, General practice/PCN, qualified 10–20 years)*

A minority of participants highlighted that there could be a lack of continuing supervision and support within their workplaces. Participants reported initial training but no ongoing support which resulted in clinicians not being aware they were doing tasks incorrectly which led to the risk of prescribing, dispensing and administration errors.

### The patient

Participants discussed that patients with mental illness could be associated with some unique challenges. During episodes of illness, patients were described as presenting to services in an agitated state. A specialist nurse described how a patients’ challenging behaviour led clinicians to prescribe differently to how they normally would have, which itself contributed to overprescribing:

*“He′s [the patient] a very difficult man to work with sometimes. He can be aggressive; he can be hostile. And I think that sometimes when he presents to services […] there is […] a penchant for services to prescribe him medications to calm him down and move him away from the service to let his regular services then pick him up. I think […] the aggression that he can present with does scare some professionals who do not know him, and as a result he can be seen as very demanding and sometimes those demands are met by inexperienced healthcare professionals*.*” (NURSE01, Community mental health services, qualified > 20 years)*

As highlighted in the previous quote, continuity is important as this pattern of response by clinicians was linked to inexperience of individual staff and was more likely to occur during periods of high demand on services. The specialist nurse quoted above goes on to describe how these illnesses may cause difficulties with patients’ understanding of their medication regimens which contributed to a prescribing error:

*“a relapse of schizophrenia […] when the individual is hearing voices and experiencing delusions and command hallucinations their, for want of a better term, grip on our collective reality is tenuous at best, and to ask them to understand the specifics of a medication regimen is wholly unrealistic considering the experiences of their subjective reality*.*” (NURSE01, Community mental health services, qualified > 20 years)*

Less than a quarter of participants discussed social factors for this patient group. Clinicians reported that some patients may have lived alone and had no family, friends or social support to assist them with regular challenges such as remembering to take medication or attend appointments which contributed to administration and monitoring errors, respectively. This patient group were also reported to lead *“chaotic lifestyles”* (GP02), i.e. complex psychosocial factors, which participants linked to patients’ difficulties in arranging and attending appointments. One GP described the various modes of communication as well as multiple attempts required to contact patients which contributed to a monitoring error (absence of tests) for an extended period of time:

*“He [the patient] was hard to get hold of. I mean obviously we can phone, we do have AccuRx which allows us to send text messages if we have got the right mobile number on the records. And if we are really not getting anywhere with those two channels we do write physical letters and post them out – which is, I think, how we eventually got him to engage – but all of that is time consuming and leads to delays while you wait to see if they get back to you*.*” (GP01, General practice, qualified > 20 years)*

Participants recognised that patients’ poor engagement with healthcare services was also on occasion a result of their symptoms of mental illness; for example, anxiety or paranoia preventing patients from travelling to monitoring appointments.

### The organisation and management

A minority of participants discussed a perceived lack of resources (e.g. staff, equipment, funding) necessary to provide effective community care services for this patient group. It was felt that resource constraints within secondary care led to patients being inappropriately discharged to the community to create space, as described by one GP which contributed to absence of monitoring:

*“…we have 8,800 patients and you cannot give the same level of intervention as maybe the secondary care team could have done had they kept him [the patient] on. And I understand they have to discharge people and create space for the ever-growing waiting list of other patients who need to be seen*.*” (GP01, General Practice, qualified > 20 years)*

A minority of participants reported that there were a high number of patients with mental illness in community care, due to discharges that were deemed to be inappropriate. This problem was exacerbated by the perceived high threshold criteria placed for patients to access secondary care mental health services, resulting in clinicians in the community being left to support patients due to referrals being rejected. This was exemplified by one GP who described a prescribing error for a patient whose complexities, they felt, warranted input from secondary services and required local political involvement to have a referral accepted into secondary services:

*“He [the patient] was presenting in A&E with self-harm, presenting in the practice, three or four times a week, so I tried to get him seen in specialist care, as often happens, you get a letter back saying, they do not meet the threshold. I made a bit of a fuss, contacted the MP [member of parliament]….” (GP03, General practice, qualified > 20 years)*.

Structure and work cultures were mentioned by a minority of participants. Clinical teams were reported to have allowed a culture to develop in which care management shortcuts became routine practice in order to meet organisational goals. These working practices were reported across multiple community services and were used because teams felt that they improved working efficiency with less consideration of whether it was beneficial to the patients and the organisation. This was outlined by one nurse who described how one clinical team had created an environment where they adopted shortcuts in order to meet patient demands. Despite education/training interventions to change their practice, staff still continued to inappropriately prescribe sedatives:

*“There were a lot of healthcare staff who had probably been there longer than the nursing staff. So there was a bit of a culture of, they were running it how they thought. And in my opinion, had too much influence over the nursing staff, which they should not have done…we [investigation team] just could not get it through to them [healthcare staff], despite the education, the support, the number of teams that were going in to educate them, they still thought that [inappropriate prescribing] was the most appropriate solution*.*” (NURSE03, Community mental health services, qualified 10–20 years)*

Policies that were not adapted for the needs of this patient group were reported as a contributory factor in medication errors. For example, participants described a policy to discharge patients without follow-up after multiple occasions of appointment non-attendance, however, this approach was deemed inappropriate for those with mental illness whose ability to attend can be a direct result of their mental illness and an indicator of condition deterioration. One general practice pharmacist described how this policy led to a complex patient being managed solely by community care when the service were ill-equipped to support the patient, which ultimately led to a colleagues’ duplication/overprescribing error of multiple antidepressants and mood stabilisers:

*“[The patient] did not attend and then [the specialist service] just discharged her. […] When we know that people with mental health problems are going to DNA [did not attend] more than the general population, is that a reason not to [keep] them in your service, because perhaps one of the signs of them becoming more unwell would be not attending appointments. So, can we just wash our hands with them and say, if they do not attend an appointment that seems, again, a very strange thing to do*.*” (PHAR11, General practice/PCN, qualified 10–20 years)*

Respondents described misplaced and inappropriate targets, which exacerbated the lack of follow-up. Policies allowed clinicians to adopt more of a checklist approach in order to meet criteria to receive payment/funding, after which there was perceived to be less impetus to follow-up patients who do not attend appointments, which resulted in a monitoring error as highlighted by one GP:

*“…our practice would send out three different invites at different points to try and get them to come in. But then if they do not respond to that, it’s what happens there, there is not really a safety net then at that point. So, in terms of QOF [Quality and Outcomes Framework], you are able to […] tick a box to say they did not respond, so then you can still get the funding. So, there’s not really any incentive at that point, unless you have got a really good GP….” (GP02, General Practice, qualified 1–3 years)*

## Discussion

To our knowledge, this is the first study to identify and examine the contributory factors to medication errors and preventable ADEs for patients with mental illness in community care settings. A total of 26 healthcare professionals discussed 43 medication errors and 12 adverse drug events from a range of settings with prescribing errors most commonly discussed. A total of six themes were identified in the occurrence of these errors/ADEs discussed. Amongst the themes, the individual (staff), the work environment, and the teams and interfaces were most commonly implicated. Highly recurring sub-themes in errors discussed included lack of knowledge accompanying diffusion of responsibility and competency/complacency of staff, workload/time and verbal/written communication between teams/across interfaces. Potentially unique patient factors were highlighted such as challenging behaviours associated with mental health symptoms, complex lifestyles and social factors. Previous patient safety research has mainly focused on physical health (43) with most mental health patient safety research exploring safety concerns such as self-harm and suicide (44). This study furthers our knowledge regarding other safety concerns by understanding the contributors to preventable medication safety incidents and acknowledges the diverse origins of risk, which will assist in proactive safety enhancement. The findings of communication issues being a key cause of errors is consistent with previous research (10), however, this study has identified a multiplicity of factors which may call for a multifaceted intervention.

The health service structure and operation increasingly places considerable responsibility and pressure on community care for patients with mental illness; in the UK, primary care treats 90% of this patient group (13). Currently in the NHS, there is a clear service structure for physical health needs which includes pathways for care, maximum waiting times and quality standards (45). In contrast, a recent review of NHS mental health standards proposed improvements, which included new waiting time guarantees and that patients should be seen by specialist teams, all to help bring parity to mental health services (46). Our findings echo these recommendations by highlighting the importance of patients with mental illness requiring care from the most appropriate service to minimise risks with their specialist medication(s). This study indicated that patients with serious mental illness, as well as patients with less serious mental illnesses, were vulnerable to inadequate care in the community and supports the NHS Long Term Plan of encouraging collaboration between GP practices and community mental health teams as a primary care network, which will hopefully bring more specialists to the forefront of care (47). Utilisation of clinical pharmacists could help provide evidence-based pharmacotherapy, improve adherence to treatment guidelines and may help reduce medication error rates in patients with mental illness (10, 48–50). Further evidence has suggested that communication and collaboration between healthcare services can be an issue (51–54). We found that care being provided exclusively by one provider or passing patients between the services introduces risk of errors, therefore a collaborative approach with a standardised and robust approach to communication may help ensure the safety of patients and their medication(s). However, a minority of general practice-based respondents also discussed the evolving complexity of health care teams working in the community and how it can lead to confusion and exclusion of professionals, as well as issues regarding continuity and autonomy. When considering future models as outlined in the NHS Long Term Plan (47), it is important to find a balance that ensures coordinated services are developed with every healthcare professional and patient in mind.

Participants described a lack of knowledge, skills and training with regards to mental illness, psychotropics and of local procedures. It has been noted in research from the UK and other countries that mental health knowledge and training is lacking amongst community healthcare professionals (55–58). In 2016, data in England highlighted that only 46% of trainee GPs undertook a training placement in mental health and 42% of practice nurses reported to have had no mental health training (59). Our findings suggest these knowledge/training gaps may accompany diffusion of responsibility, as some healthcare professionals may view this specialty beyond their remit and therefore consider themselves to have less impetus to acquire this knowledge, as patients with mental illness are seen as the responsibility of specialist services. This finding may raise the need for further exploration of the education and training of these professionals as knowledge gaps about, and attitudes towards, mental healthcare may originate in early education (60–62). These findings indicate that it is important that healthcare professionals learn how to work together early in their careers with different disciplines to encourage better collaborative efforts, especially as there is a new integrated service structure as per the NHS Long Term Plan (47). It has been reported in the UK and internationally that medical students can view psychiatry with a negative attitude and that it is not considered equal to physical health specialties, which can deter students from pursuing interest in the specialty (61, 63). In the UK, data from 2021 highlighted that 80% of undergraduate pharmacy students are taught neuropharmacology (64), which may explain why the majority of pharmacists reported high levels of knowledge and confidence regarding psychotropic medications during interview. However, the undergraduate pharmacy curriculum has less emphasis on communication skills in the context of mental healthcare (64) and some medical students report no substantive mental health content until the latter years of the degree (65). Recommendations for educational reform include strong neuropharmacology knowledge across all healthcare disciplines and improved communication skills regarding mental health, which should be incorporated into undergraduate and foundation training programmes (64, 66). The use of interprofessional education may help achieve this, as there are shared learning objectives which encourage collaboration and communication with the potential to improve patient safety (67).

Respondents reported some unique challenges that this patient group present with such as symptoms creating behavioural challenges and lifestyle/social factors. The finding of patient symptoms/behaviours creating barriers is not dissimilar from the UK hospital context (23), however, patient lifestyles and social factors appears to be an issue of equal importance whilst these patients are in the community. It was also evident from the findings that a blanket approach to the application of healthcare policies could disadvantage patients with mental illness, who can present with additional challenges, therefore adaptations to policies that consider the specific needs of this patient group are recommended, for example, discharge processes and barriers to accessing services. Previous research has highlighted that patients can provide an important insight into medication safety incidents within the community (68), and arguably greater research attention is now needed for patients with mental illness due to potential unique contributory factors highlighted in this study. It is crucial to ensure research in this area is patient/carer led, as it will help to ensure future intervention research considers their needs (69, 70). Future research could qualitatively explore this topic from the patient and carer perspective to help gather further detailed insight from their perspective and create a holistic picture in conjunction with the findings from this study (69, 71). Having patients take an active role in healthcare safety is also a key target for the NHS and World Health Organisation (1, 72).

### Strengths and limitations

The analytical and error frameworks used for data analysis and interpretation are well established and have been used in other health and medication error research (20, 42). A selection of transcripts were independently read by members of the research team to verify any themes emerging from the data and the final set of themes were presented to a patient advisory group and verified. Participants were asked to talk about errors that they made themselves or had direct first-hand knowledge of why an error occurred to ensure contributory factors were not based on generalised views. However, the majority of errors discussed by participants were made by another clinician which raises the possibility that they would not have had full insight into what other people were thinking at the time of the error, even if that person had discussed the incident with them. Future research should explore accounts from professionals who made the error. There may have been a degree of social desirability bias whilst participants recalled incidents due to the sensitive nature of the topic; participants may have modified accounts to make them sound less serious, which may have limited data availability. As participants discussed accounts that happened in the past, there is a potential for recall bias. However, this risk was minimised by encouraging participants to discuss errors that were significant in their practice, as well as allowing participants to use resources such as emails, medical records etc. to aid in the recall of events (these were not shared with the research team). Self-serving attribution bias may also have been a possibility as respondents may have deflected blame onto others to place themselves in a more positive light (73, 74). Previous research has highlighted limitations of remote interviews such as lower quality data and difficulty building rapport (75). This was mitigated by following an interview schedule which helped ensure consistency of data being obtained and the use of a webcam to allow the interviewer to respond to visual cues. There was underrepresentation of some professional groups, for example, psychiatrists. However, there was representation from all eligible professional groups and data collection continued beyond the point of saturation. The research teams professional networks mostly consisted of contacts within the Northwest of England which may impose regional bias. However, there were no differences in accounts between regions and there was representation of professionals from other regions in the UK, as snowballing was encouraged to reach participants that the research team did not have contact with. Finally, error accounts were from the viewpoint of one professional within a healthcare chain and there is the possibility that some details were missed in the series of events, which may have affected their awareness about local versus wider system factors.

## Conclusion

This is the first study to identify and examine in-depth the contributory factors of medication errors and adverse drug events for patients with mental illness in community care from the perspective of healthcare professionals. The findings highlight that the causes of errors are multifactorial but there were some unique to this patient group. Clinicians’ lack of knowledge regarding psychotropic medication/mental illness accompanied diffusion of responsibility; teams were unclear with responsibility and communication; and patient lifestyle/social factors were not reflected in policy and systems. There is a need for further examination of education in neuropharmacology and communication skills amongst healthcare professionals in order to foster improvement. Future healthcare policy should seek to improve discharge processes and barriers to accessing services. Future research should explore this topic from a wider geographical distribution and sample of healthcare professionals to confirm these findings. There is also a need to explore the contributors to preventable medication safety incidents in more detail from a patient perspective to help build a holistic picture of this topic. The findings from this study can help improve medication safety for this patient group by guiding local healthcare changes, as well as informing future improvement intervention research efforts.

## Data availability statement

The original contributions presented in the study are included in the article/Supplementary material, further inquiries can be directed to the corresponding author.

## Ethics statement

The studies involving humans were approved by the University of Manchester Research Ethics Committee 1 (reference 2022-13735-23555). The studies were conducted in accordance with the local legislation and institutional requirements. The participants provided their written informed consent to participate in this study. Written informed consent was obtained from the individual(s) for the publication of any potentially identifiable images or data included in this article.

## Author contributions

MJA: lead role in conception, design, data collection, analysis, interpretation, drafted, revised and approved the manuscript. PJL, DLP, and RNK: conception, design, analysis, interpretation, revised and approved the manuscript. All authors contributed to the article and approved the submitted version.
