# Supplementary material for: unDerstandIng the cauSes of mediCation errOrs and adVerse drug evEnts for patients with mental illness in community caRe (DISCOVER): a qualitative study
Source: Front Psychiatry. 2023 Dec 7;14:1241445. doi: 10.3389/fpsyt.2023.1241445 (PMC10746165; doi:10.3389/fpsyt.2023.1241445)
Supplement: Supplementary file 1 [file Table_1.DOCX]

**
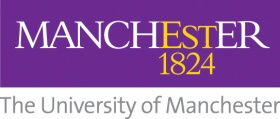
UnDerstandIng the cauSes of mediCation errOrs and adVerse drug evEnts for patients with mental illness in primaRy care (DISCOVER): a qualitative study**

**Interview schedule**

The purpose of the interview is to explore the causes of medication errors which cover the domains of prescribing, monitoring, dispensing, and administration and also the causes of preventable adverse drug events which are harm caused to a patient as the result of a medication error. We want to investigate these errors/incidents in relation to medication used by patients with mental illness in primary care. The medication involved can be either psychotropic or non-psychotropic.

Your right to confidentiality is protected and assured at all times throughout the study. Confidentiality will only be breached in circumstances where participants disclose potential malpractice or misconduct as outlined in the participant information sheet. Any information provided in this interview that is analysed and reported will not allow anyone to recognise you. Any patient or colleague details such as names are not required; if you do mention any such details then they will be removed from all records.

The interview will last approximately 30-60mins and will be video/audio recorded using Microsoft Teams/Zoom unless you do not wish this then we can use a telephone instead. You can request for recording to be stopped at any point during the interview. The recordings will be stored on a secure drive which is password protected and only accessible to the research team.

I will start the interview by getting some details about you and your professional background, then I will ask you about one or more medication errors/adverse drug events that you were involved with and asked to think about before attending this interview. I will then ask you about ways the incident could have been prevented from your point of view. Finally, I will finish by asking some questions in case we missed anything and allowing time for you to ask questions.

Do you have any questions before we start the interview? – start recording after Q&A

**Part One – Introduction & Background**

I just want to start by asking you some questions about you and your professional background.
Please could you tell me your…

- Name
- Educational/training background (primary degrees & any relevant psychiatric and/or medication training)
- Number of years qualified/practicing
- Number of years working in primary care
- Current job role/title
- Primary care sector currently working in (if no longer working in primary care which primary care sector did you work in within the last 5 years)
- Area of the UK working e.g. country and region

**Part Two – the medication error/adverse drug event**

In the email I sent to you, I asked if you could talk about one or more medication error(s) (which could be related to either prescribing, monitoring, dispensing or administration) and/or episode(s) of preventable harm caused to a patient as the result of medication errors which you were involved with.

- Type of incident – medication error and/or adverse drug event
- Type of error(s) – skip if participant just wants to discuss an ADE
  - Prescribing – omission, no indication/wrong drug for indication, duplication, incomplete prescription, drug allergy, inappropriate drug choice, inappropriate dose, frequency, quantity, incorrect route, incorrect formulation, unnecessary drug use (under/overprescribing), inappropriate abbreviations, illegible
  - Monitoring – inadequate follow up, absence of tests, failed to review regime for appropriateness and detection of problems, failed to use clinical tests (e.g. bloods) for adequate assessment of patient response
  - Dispensing – wrong patient, wrong drug, wrong dose, wrong formulation, wrong quantity, wrong label (directions), expired, omission
  - Administration – dose, frequency, duration, formulation, omission, route, wrong patient
- Was more than one error type involved in the incident – if only mentioned one previously
- Medication involved – drug name, strength, formulation
- What was being treated – diagnosis and condition severity
- Did the error reach the patient?
  - What harm was caused to the patient as a result of the error/ADE?
  - Was any additional medical treatment necessary?
- How was the error/ADE discovered?
  - Was the error picked up at the source or further down the chain? E.g. another clinician or the patient reported it

Circumstances of error/adverse drug event

- Time, date, location
- Physical and mental state at time of error/adverse drug event
- Other people involved – their job role
- Workload at the time
- Was there any support/advice available from more experienced colleagues?
- Patient situation – under a consultant or treated solely in primary care, Your patient? Previous contact with patient? Patient Complexity?

**Refer to interview tips on other page**

**Part Four – Conclusion**

- Is there anything additional you want to discuss?
- Is there anything you want to add or go back to?
- Do you have any questions?

Stop the recording

Thank you so much for giving up some of your time to take part in this interview study. Your information and contribution has been extremely valuable in helping us to address our study aim. Please feel free to contact me if you have any questions or other issues. Your contact details will be used to organise your high street voucher compensation payment - confirm contact details. If you signed the consent form requesting a copy of the results and/or wishing to be contacted about future research opportunities, then your details will be held on record in order to contact you in the future.

**Part Three – causes of the error/ADE**

- Identify immediate active failure: slip (not doing what you are meant to do), lapse (forgetting), mistake (rule or knowledge based) or violations (intentional failures)
- Explore contributory factors: communication, work environment, task support, knowledge, training/experience, support/social issues, procedures, team working, organisational issues, interface care (e.g. between MH services and primary/secondary care)
- Knowledge/opinion towards procedures and medication
- Evidence of multiple contributory factors involved, how do they link together?
- Identify any mental health specific contextual factors?

Reflection

- Could anything have prevented the error/ADE?
  - What factors were prominent?

**Interview tips**Difficulty recalling incident – consider an incident that had a profound effect on them and their practice

- Could you tell me a bit more about that?
  - Could you give me a more detailed description of what happened?
  - Tell me what you are thinking about
  - You mentioned… – what do you mean by that?
  - You mentioned… before, could you talk a more about that?
  - Why did you hesitate just then?
